# The value of recognizing suspect diagnoses in the triple diagnosis of giant cell tumor of bone

**DOI:** 10.4103/0019-5413.32038

**Published:** 2007

**Authors:** Mrinalini Kotru, Navjeevan Singh

**Affiliations:** Department of Pathology, University College of Medical Sciences and GTB Hospital, Delhi - 110 095, India

**Keywords:** Bone tumor, giant cell tumors of bone, GCT, orthopedic pathology, triple diagnosis.

## Abstract

Giant cell tumor (GCT) of bone is the most frequently over-diagnosed neoplasm in orthopedic pathology because giant cells are a common component of many neoplastic and nonneoplastic conditions of bone. Triple diagnosis, requiring substantial individual and collective inputs by orthopedic surgeons, radiologists and pathologists, is the preferred method for the workup of patients with suspected bone neoplasms. At each stage in triple diagnosis, deviations from the typical must be regarded as clues to alternate diagnoses: the greater the deviation, the more a diagnosis of GCT must be considered suspect. A suspect diagnosis must trigger renewed analysis of the available data and a diligent search to exclude alternate diagnoses.

This review lists suspect diagnoses of GCT with a brief overview of each.

Orthopedic pathologists were among the earliest to emphasize the importance of a combined approach to tumor diagnosis. Essentially, this means that the diagnoses based on a) clinical data, b) radiological and other imaging analysis and c) pathologic evaluation must, individually and collectively, contribute substantially towards establishing the correct diagnosis.

A brief listing of the clinical and radiographic features of the case at hand, the location of the lesion: whether epiphyseal, metaphyseal or diaphyseal and the clinical and radiological diagnosis or a list of differential diagnoses, must therefore be a minimum requirement for submitting tissue for histopathological analysis. Armed with this information the pathologist is expected to make an intelligent assessment of the histopathology to come to a diagnosis.

## THE CLASSIC GIANT CELL TUMOR OF BONE

The many elaborate descriptions of giant cell tumor of bone in the literature^1^–^3^ scarcely require repetition. Briefly, the classic GCT affects the mature skeleton with closed epiphyseal plates, most commonly in the third decade of life. Serum chemistries are typically normal. Located near the articular ends of the lower end of the femur or upper end of the tibia, the epiphysis is invariably involved by the radiographically lytic lesion. In this clinical and radiological setting, the histopathological picture of diffusely dispersed osteoclast-like giant cells in the characteristic stroma is diagnostic of giant cell tumor.

**Figure 1:**
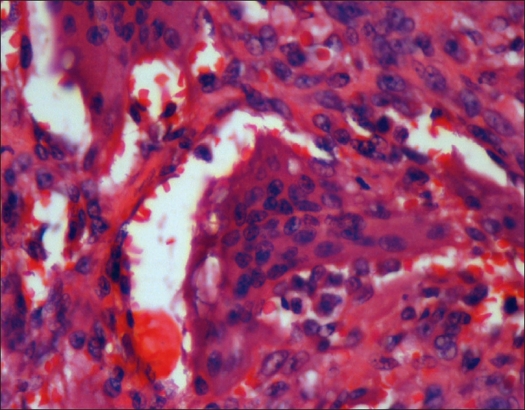
Large multinucleate giant cell in characteristic mononuclear stroma. Note the resemblance of the nuclei of the stromal cells to giant cell nuclei. (Hematoxylin and eosin, ×400)

The morphology of the background population of mononuclear cells is crucial to the diagnosis. Essentially these are round, oval or polygonal in shape with nuclei closely resembling those in the giant cells [Figure 1]. Sometimes the mononuclear cells are spindled with varying amounts of eosinophilic cytoplasm; these are less diagnostic and efforts to examine more tissue to look for the diagnostic cells must be made. Mitoses may be abundant in the mononuclear cells, but do not predict behavior of the tumor.

The giant cells in GCT often contain a large number of nuclei, sometimes over a hundred, a feature rarely seen in other osseus neoplasms. It is thought that giant cells form by fusion of the mononuclear cells. Supporting this concept is the fact that mitosis is never seen in the giant cell nuclei. Osteoclast-like giant cells in GCT are thought to derive from a monocyte-macrophage lineage. Besides others,^4^ they have been shown to express the macrophage marker HAM-56.^5^ The stromal cells are thought to originate from mesenchymal stem cells.^6^

Many neoplastic and nonneoplastic lesions of bone may contain varying proportions of giant cells. Probably the best way to minimize the risk of misdiagnosis is to consider any variation from the classical as a clue to an alternate diagnosis. Identifying a suspect diagnosis is thus an important first step towards the correct diagnosis.

## DIFFERENTIAL DIAGNOSIS

A brief consideration of the differential diagnosis of each of these situations is presented below [Table 1].

**Table 1:** Suspect diagnosis of giant cell tumor of bone

By clinical features and site	By radiographic appearance	By gross and microscopic features
GCT in an immature skeleton with open epiphyseal plates GCT occurring *de novo* in a patient older than 55 years GCT in a patient with elevated serum calcium GCT near articular ends of long tubular bones (other than around the knee joint): i) distal radius, ii) proximal femur, iii) proximal humerus and iv) distal tibia GCT involving the flat bones other than the sacrum and the pelvis GCT of the craniofacial (particularly the jaw) bones, except in a patient with Paget's disease GCT of the small bones of the hands and feet GCT of the vertebrae above the sacrum Multicentric GCT	GCT not involving the epiphysis GCT with perilesional sclerosis GCT with periosteal calcifications (sunburst, onionskin, Codman triangle)	GCT in the presence of uninvolved or open epiphysis GCT with diffusely permeative growth pattern GCT with reactive sclerosis GCT with abundant matrix production GCT with cartilage in unfractured tumors GCT with giant cells in clusters

### I) BY CLINICAL FEATURES AND SITE

#### GCT in an immature skeleton with open epiphyseal plates

Most GCTs occur in patients older than 20 years of age i.e., after the closure of epiphyses. The peak incidence is in the third decade. Very rarely, GCT occurs in younger patients.^7^ In such cases it is seen in late teenage involving the bones of hands and feet. Osteosarcoma with prominent giant cells, one of the most serious diagnostic pitfalls in orthopedic pathology, must enter the differential diagnosis in patients with immature skeleton.

#### GCT occurring *de novo* in a patient older than 55 years

*De novo* tumors in older patients are more likely to be malignant tumors other than GCT. Occasionally, a primary malignant GCT, which may have sarcomatous areas, can be encountered. Spontaneous malignant transformation of GCT in older patients has also been described.^8^ There appears to be no reliable way of knowing which of these tumors will undergo malignant change.^9^

#### GCT in a patient with elevated serum calcium

Elevated serum calcium must suggest brown tumor of hyperparathyroidism^10^ and giant cell reparative granuloma, which regardless of its location, is histologically indistinguishable from it. Serum calcium, phosphate and alkaline phosphatase levels should be determined. Serum parathyroid hormone levels should be determined when calcium levels are at the upper limits of normal to exclude normocalcemic hyperparathyroidism.

**GCT near articular ends of long tubular bones (other than around the knee joint):** (i) distal radius, (ii) proximal femur, (iii) proximal humerus and (iv) distal tibia.

More than 50% GCTs occur in the region of the knee.^3^ If all other parameters are in agreement, the diagnosis is most likely to be correct at this site than at any other. Giant cell tumor has been reported in the distal radius, proximal femur, proximal humerus and distal tibia in reducing order of frequency compared to around the knee joint. Therefore, at these sites the diagnosis of GCT must be considered increasingly suspect, until proved otherwise.

#### GCT involving the flat bones other than the sacrum and the pelvis

When GCT does occur in the flat bones, the sacrum and the pelvis are favored sites, albeit with the caveat that these are relatively rare sites.^11^

#### GCT of the craniofacial (particularly the jaw) bones, except in a patient with Paget's disease

Most Paget sarcomas are osteosarcomas. GCT very rarely arises in patients with Paget's disease, when it may involve the craniofacial bones.^12^ The distribution of GCT tends to parallel the distribution of uncomplicated Paget's disease. It pays to remember that most giant cell lesions of the jaw are giant cell reparative granulomas^13^,^14^ in which the giant cells tend to have fewer nuclei and be aggregated around areas of hemorrhage;

#### GCT of the small bones of the hands and feet and GCT of the vertebrae above the sacrum

Giant cell tumor of the small bones of the hands and feet^15^ and of the vertebrae above the sacrum^16^ are very rare. Most giant cell lesions of the small bones of the hands and feet are reparative granulomas. Those of the vertebrae are aneurysmal bone cysts which, unlike GCT, involve the posterior arch and spinous processes.

#### Multicentric GCT

Although rarely, multicentric GCTs^17^ have been described, however, multifocal giant cell lesions are more likely to be brown tumors of hyperparathyroidism, a question easily settled by serum chemistries and alkaline phosphatase determinations.

### II) BY RADIOGRAPHIC APPEARANCE

**GCT not involving the epiphysis**

**GCT with perilesional sclerosis**

**GCT with periosteal calcifications (sunburst, onionskin, Codman triangle)**

The interpretation of radiographic features is outside the scope of most pathologists' work, emphasizing the reliance that must be placed on the radiologists' input.

### III) BY GROSS AND MICROSCOPIC FEATURES

#### GCT in the presence of uninvolved or open epiphysis

Involvement of the epiphysis can be ascertained by the pathologist only in resection or amputation specimens. The pathologist must be informed of radiographic evidence of epiphyseal involvement when curettings are submitted for histopathological examination.

#### GCT with diffusely permeative growth pattern

Diffusely permeative radiographic and histopathological growth patterns are indicative of malignant tumors, notably osteosarcoma,^18^ which often infiltrate considerably into surrounding tissue. Exceptions occur when the metaphyseal region of a GCT is sampled: a permeative growth pattern, corresponding to the ill-defined margin seen radiographically, may be seen on microscopic examination. Identification of the region of the tumor that has been sampled - essential for correct interpretation - can be a problem with curetted material. When dealing with resection specimens, specimen radiography as an aid to tissue sampling is beneficial.

#### GCT with abundant matrix production

About half of all GCTs exhibit reactive osteoid and woven bone at the advancing edge of the tumor. This must be distinguished from the tumor osteoid of osteosarcoma. The clues to the diagnosis lie in the radiographic analysis and in the morphology^3^,^19^ of the stromal cells. The background mononuclear cells of GCT are predominantly round, oval or polygonal in shape and many are indistinguishable from normal histiocytes. The nuclei closely resemble those within the giant cells. Pleomorphism and atypical mitotic figures must be considered indications to exclude osteosarcoma and to embed and section all the available tissue, particularly if the radiography is atypical and the skeleton immature.

#### GCT with reactive sclerosis

Reactive sclerosis should suggest nonossifying fibroma^20^ (NOF). Nonossifying fibroma occurs in the metaphysis of younger patients. Spindle-shaped stromal cells with fibrosis and aggregates of foamy macrophages may be seen focally in GCT and extensively in NOF. Review of radiological and clinical features should allow easy differentiation.

#### GCT with cartilage in unfractured tumors

Cartilage may be seen in fractured GCT as a manifestation of healing fracture.^3^ Healing fracture is a diagnostic pitfall for many osseus and cartilaginous neoplasms of bone.

Careful evaluation for histopathological signs of organization and zonation in healing fracture must be made. Unless fracture can be supported by radiographic evidence, a diagnosis of GCT must not be made in the presence of cartilage.

#### GCT with giant cells in clusters

In general, uniform distribution of large giant cells in the appropriate stromal background is characteristic of GCT. Smaller giant cells, with fewer nuclei, aggregate around areas of hemorrhage in reparative granuloma^21^ and brown tumor of hyperparathyroidism. In other situations giant cells present focally should be a clue to an alternate diagnosis such as aneurysmal bone cyst.

#### Behavior

GCT is an aggressive lesion with high degree of local recurrence and malignant potential. Despite the advocacy of several grading systems,^22^,^23^ if true sarcomas are excluded, there appears to be no reliable way of knowing which of these tumors will undergo malignant change. When transformation does occur, on an average, mean age of transformation seems to be seven years. The average age of transformation reduces further if the GCT has been previously treated with irradiation.

Benign metastasizing GCT is an uncommon, poorly understood phenomenon where otherwise typical GCTs exhibit pulmonary metastases. These may be present at the time of initial presentation or be detected up to several years later. The metastatic lesions are histologically identical to the primary tumor.^24^ Histopathological examination of the pulmonary lesion and review of the tissue removed at the time of the initial diagnosis must be carried out to exclude either malignant change in a GCT or a sarcoma with giant cells.

## CONCLUSION

Successful therapy is critically dependent on accurate diagnosis. Recognition of suspect diagnoses is important in avoiding mis-diagnosis of giant cell lesions of bone, of which GCT is but one. For maximal benefit this concept must be utilized in all three arms of the triple diagnosis.
